# The Ongoing Incorrect Use of Caucasian in Medical Research

**DOI:** 10.1089/heq.2025.0014

**Published:** 2025-03-21

**Authors:** Jamila K. Picart, Sidra N. Bonner, Gurjit Sandhu

**Affiliations:** ^1^ Center for Healthcare Outcomes and Policy, University of Michigan, Ann Arbor, Michigan, USA.; ^2^ Institute for Healthcare Policy and Innovation, University of Michigan, Ann Arbor, Michigan, USA.

**Keywords:** race, health equity, health services research

## Abstract

As medical research continues to promise the advancement of health equity, it is called to address its incorrect and ongoing use of the term “Caucasian.” The term “Caucasian” has persisted in medical research despite its entanglement with beliefs of race as a biological factor. To continue to advance efforts in addressing health disparities and achieving health equity, researchers are called to use accurate racial and ethnic terminology.

Increased attention to the disparate impacts of race and ethnicity on medicine and health have begun to close the gap in health inequities. In 2020, health services researchers were called to re-address the manner in which we discuss and analyze race and health inequities.^1^ Yet, as we continue to unravel the relationship between medicine and race and ethnicity, we still have shortcomings to address that require our immediate attention. One such shortcoming is the ongoing use of “Caucasian” in published medical research. “Caucasian” is an outdated and discredited term often used instead of the accurate term “White.” While there has been much effort by major medical journals to remove the term “Caucasian” from their publications, a search in PubMed of articles published in 2023 demonstrates over 1500 articles printed the term.^2^

Our communal shortcoming in using “Caucasian” instead of “White” has become glaring given recent revisions to federal policy guiding the accurate reporting of race and ethnicity terms. As of March 2024, the U.S. Office of Management and Budget submitted a directive to revise the terminology the federal government uses to report race and ethnicity. One revision within the policy, for the first time in U.S. history, disaggregates ethnicities, such as “Middle Eastern and/or North African” and “Hispanic/Latino” from the racial category “White.”^3^ Prior federal collection standards for the racial category “White” equated the term with previously used definitions for “Caucasian.” In concordance with federal policy and to ensure accuracy in scientific research, medical research is called to stop using the term “Caucasian.”

The historical origins of the term “Caucasian” imbue the term with inaccurate beliefs that race has biological significance. In the 18th century, Carl Linnaeus, a botanist and the “Father of Taxonomy,” was determined to create a classification system for the human species. He created four divisions of humans corresponding to the four known continents: Homo americanus, Homo europaeus, Homo asiaticus, and Homo africanus.^4^ In this system, he ascribed to the different groups of humans not only differences in their physical characteristics, such as race, but also differences in their aptitude and morality. Linnaeus was not a proponent of scientific racism; however, Linnaeus’ work has still been incorrectly used as a foundation for scientific racism, or the belief that racial identity implies a certain superiority/inferiority or aptitude.

Ongoing entanglement of human race with sentiments of superiority or increased aptitude continued with German anatomist Johann Blumenbach’s work which included creating the term “Caucasian.” In the late 18th century, Blumenbach, attempting to advance the science of human classification, examined the skulls and specimens of humans from different geographical regions. From his work, he concluded there should be five divisions of human: “Caucasian,” “Ethiopian,” “Oriental,” “Malay,” and “red.”^5^ The naming system mostly based on the regions from which he took samples. In his work, Blumenbach crafted a narrative framing the “Caucasian” race, those originating from the region of Caucasus, as most closely resembling the ideal human. Blumenbach’s work has also been used to advance scientific racism.

While the beliefs and principles of scientific racism were not held by Linnaeus or Blumenbach, the misrepresentation of their work to bolster scientific racism lingers. Science largely accepts that race is a social construct. We no longer perpetuate the misnomer that the color of someone’s skin or their geographic origins are an explanation for their physical strength, intelligence, or morality. While the biological basis to race may be discredited, the beliefs can linger in society by the persistent use of terms associated with their foundation, such as the use of “Caucasian.”

Although medical research does not intend to employ scientific racism, the term “Caucasian” is still printed in major scientific journals. Several theories exist to explain the persistent and inappropriate use of “Caucasian.” First, “Caucasian” may linger from longstanding use by the U.S. federal government.^6^ Blumenbach’s manufactured races created the basis for racial classification in the U.S. Second, “Caucasian” may represent a protective euphemism.^7^ In medical research, using “Caucasian” instead of “White” may be seen as a path to focus on the science and soften discussions of racial inequalities in medicine. This desire to “tread lightly” is supported by recent surveys in the United States suggesting that discussions on race and race relations have been getting worse.^8^ However, the term “Caucasian” bears real weight on people from historically disadvantaged racial and ethnic identities given its historical background.

The removal of “Caucasian” from our research promotes opportunity to advance health equity for historically under-researched populations. For example, “Caucasian” in its standard use includes Middle Eastern and North African populations in its aggregation. Yet, there is a growing collection of literature that those who identify as Middle Eastern and North African in America do not identify as White.^9^ Furthermore, objective data highlight differences in health, socioeconomic status, and social patterns in Middle Eastern and North African populations from those who identify as White.^10^ This is just one example of the many groups that benefit from the disaggregation that would occur with using accurate racial and ethnic terminology.

Therefore, we recommend strategies for journals and researchers to adopt that would encourage the use of accurate racial and ethnic terminology to support productive discussions of health inequities including:

*Standardize guidelines across health and medicine funding sources and journals related to accurate use of racial and ethnic terms.* Clear standardized guidelines across journals and funding sources would benefit authors and could be incorporated into research design without concerns for journal/funding specificity. Reviewers would also benefit from a consistent reference for rigorous evaluation of the use of appropriate racial and ethnic terms in article review. The *Journal of the American Medical Association* has clear guidelines for accurate reporting of race and ethnicity.^11^ Also, the National Institute for Health publishes clear and specific guidance on how to report race and national origin for the medical research community.

*Authors should be transparent in the limitations of their data and adapt best practice guidelines when reporting race and ethnicity.* Equally important to the creation of a conceptually or methodologically sound article is the accurate reporting of race and ethnicity data. Reporting the missingness of data or remarking when data is imputed (common in Medicare data) is important to understanding the research, its limitations and implications. Historical data may not follow current reporting guidelines. Authors should align historical data with current race and ethnicity reporting guidelines. When this is not possible, authors should disclose reasons for deviation from current race and ethnicity reporting guidelines.

*Authors should be knowledgeable of the health inequities and disparities relevant to their area of investigation.* Knowledge or expertise in an area of medical or health research should be expected to include how it may disparately impact subsets of the population, including racial and/or ethnic populations. Understand these disparate impacts can be driven by cultural, environmental, social, and political factors. Include members of these populations in your research team to drive research that is meaningful and impactful.

As medical researchers strive to address health inequities, we have been unintentionally using incorrect terminology to report race and ethnicity. To continue to close the gap in health inequity, the medical community is urged to stop using the term ‘Caucasian.”

## Abbreviations Used

US: United States
